# Immigration-related inequalities across the perinatal mental health care pathway among new parents in Sweden: a population-based cohort study

**DOI:** 10.1136/bmjopen-2026-122722

**Published:** 2026-08-17

**Authors:** Roxanne Kovacs, Nawi Ng, Gustav Kjellsson

**Affiliations:** 1 Department of Economics, University of Gothenburg, Gothenburg, Sweden; 2 School of Public Health and Community Medicine, University of Gothenburg, Gothenburg, Sweden

**Keywords:** MENTAL HEALTH, Postpartum Period, Depression, Postpartum

## Abstract

**Abstract:**

**Objectives:**

To examine immigration-related inequities across the perinatal mental healthcare pathway, including differences in case detection, treatment uptake and timeliness of care, among first-time parents in Sweden.

**Design:**

Population-based cohort study using linked administrative register data.

**Setting:**

The three largest Swedish regions (Stockholm, Västra Götaland and Skåne) covering approximately 55% of the national population.

**Participants:**

All first-time parents with a live first birth between 1 January 2008 and 31 December 2018 residing in the study regions (404 043 mothers and 391 068 fathers).

**Exposure:**

Individuals’ country of birth (born outside vs inside the Nordic countries).

**Main outcome measures:**

We examine immigration-related inequalities across the perinatal mental healthcare pathway, including case detection (issues recorded a year postpartum, with no diagnoses/treatments in the previous year), treatment uptake (antidepressants and/or psychotherapy) and timeliness of care (weeks from diagnosis to treatment).

**Results:**

In the 12 months postpartum, 7.2% of mothers and 4.5% of fathers had common perinatal mental health conditions requiring clinical management. Parents born outside the Nordic countries had substantially lower detection rates: −5.3 percentage points for mothers (95% CI −5.8 to −4.9; 59% below the Nordic-born baseline of 9.0%) and −2.5 percentage points for fathers (95% CI −2.8 to −2.3; 48% below the Nordic-born baseline of 5.2%). Conditional on diagnosis, immigrant mothers and fathers were 12.1 and 7.6 percentage points less likely to receive treatment, respectively. Differences in time from diagnosis to treatment were small.

**Conclusions:**

Immigration-related inequalities were observed across multiple stages of the perinatal mental healthcare pathway. As previous evidence suggests similar or higher levels of perinatal mental health problems among immigrant parents, the observed differences are unlikely to reflect lower need for care. Findings instead point to inequalities in access and use of perinatal mental healthcare and highlight the need for more equitable screening, assessment and follow-up.

**STRENGTHS AND LIMITATIONS OF THIS STUDY**

- Large population-based cohort including all first-time parents in three major Swedish regions, with linkage of national and regional registers covering primary care, specialist care and dispensed prescriptions.
- Examination of sequential stages of the care pathway (detection, treatment uptake and timeliness), allowing identification of where inequalities arise.
- Use of individual-level socioeconomic measures and municipality fixed effects to reduce confounding by area-level and compositional factors.
- Outcomes based on recorded diagnoses and dispensed treatments in administrative data; undiagnosed or untreated conditions are not captured.
- Definition of ‘new’ mental health issues based on a 12-month window, which may misclassify recurrent or chronic conditions outside this period.

## Introduction

 The perinatal period is critical for parental mental health. Common perinatal mental health conditions, such as depression and anxiety, are prevalent in the year following childbirth and impose a substantial burden on parents, including persistent distress and impaired functioning.^1^ These conditions are also associated with poorer cognitive, emotional and behavioural outcomes in children, including increased risk of behavioural problems, emotional difficulties and delays in cognitive development.^2^ Early identification and timely treatment are therefore central components of perinatal healthcare systems.

The perinatal setting provides a particularly informative context in which to assess inequalities, as mothers are offered routine screening for depressive symptoms approximately 6–8 weeks postpartum.^3^ In principle, this should facilitate case identification and access to treatment. We focus on inequalities based on individuals’ country of birth, a widely used indicator of social stratification in Sweden and other Nordic countries.^4^ Prior survey-based studies report higher levels of postpartum depressive symptoms among immigrant women compared with native-born women.^5 6^

A large and growing literature documents socioeconomic and migration-related inequalities in perinatal mental health. However, most existing studies focus on single endpoints, often screening for perinatal depression,^7^ and many rely on neighbourhood-level measures of socioeconomic context rather than capturing individual characteristics.^8 9^ This limits insight into how disparities arise across stages of care and risks ecological misclassification. This study is among the few to examine a sequence of outcomes along the care pathway—from case detection to treatment among those with recorded need and to timeliness of treatment—consistent with treatment cascade frameworks in perinatal depression research.^10^ By using individual-level socioeconomic data, we provide a more accurate assessment of immigration-related inequalities in perinatal mental healthcare.

The study also improves on existing registry-based research in terms of data coverage and case ascertainment. Most registry-based studies on perinatal mental health from the Nordic countries rely primarily on tertiary and specialist psychiatric registers, excluding the majority of cases, which are treated in primary care—leading to correspondingly low prevalence estimates.^11 12^ By integrating primary, specialist and hospital data, we capture detected perinatal mental health conditions more comprehensively than previous work.

Finally, whereas most existing studies focus exclusively on mothers,^13^ we analyse both mothers and fathers within a unified framework. This enables direct comparison of inequalities in case detection, treatment and timeliness across parents.

The aim of this study is to examine immigration-related inequities (based on individuals’ country of birth) in case detection, treatment and timeliness of perinatal mental healthcare among first-time parents in Sweden.

## Methods

### Study design and setting

The study was conducted in the three largest Swedish regions—Stockholm, Västra Götaland and Skåne—which together comprise approximately 5.5 million residents (around 55% of the national population). This was a population-based cohort study using linked national and regional administrative registers. The cohort included first live births occurring between 1 January 2008 and 31 December 2018. Follow-up extended 12 months from the date of childbirth. The study period ends before 2019, to avoid confounding due to disruption during the COVID-19 pandemic.

Sweden has a tax-funded universal healthcare system with high levels of coverage and minimal financial barriers to access.^14^ Antenatal care and child health services are provided within primary care and are free of charge. Nearly all pregnant women participate in routine antenatal care, and uptake of postnatal and child health services is high.

Maternal and child health services in Sweden are delivered through antenatal care clinics (Barnmorskemottagning) and child health centres (Barnavårdscentral). There is no standardised national programme for screening fathers for postnatal depressive symptoms. As part of routine postnatal follow-up, mothers are offered screening for depressive symptoms approximately 6–8 weeks after childbirth, commonly using a validated instrument such as the Edinburgh Postnatal Depression Scale.^15^ Individuals with elevated symptom scores may be referred for further assessment or treatment within primary care or to specialist psychiatric services according to regional referral pathways. Primary care physicians are responsible for diagnosing and managing common mental disorders, including depression and anxiety, and may initiate pharmacological treatment or provide psychological therapy. Referral to specialist psychiatric care typically occurs in cases of greater severity or complexity.

The paper is organised in accordance with the Strengthening the Reporting of Observational Studies in Epidemiology guidelines (STROBE) for cohort studies and the Reporting of studies Conducted using Observational Routinely-collected Data extension for studies using routinely collected health data.

### Participants

Eligible participants were all first-time mothers and fathers residing in the study regions at the end of the year of childbirth, with a first live birth between January 2008 and December 2018. Parents and children were identified using the Multi-Generation Register maintained by Statistics Sweden. First-time parents of adopted children and parents who gave their child up for adoption were excluded.

### Data sources

National register data were used to obtain information on inpatient and specialist outpatient diagnoses from the National Patient Register, dispensed prescriptions from the Prescribed Drug Register and sociodemographic characteristics from registers maintained by Statistics Sweden, including sex, education, income, municipality of residence and country of birth. Regional healthcare registers from Stockholm, Västra Götaland and Skåne were used to identify primary care contacts, International Statistical Classification of Diseases and Related Health Problems 10th Revision (ICD-10) diagnoses and clinical procedure codes (klassifikation av vårdåtgärder, KVÅ). The inclusion of regional primary care data allows us to capture the majority of common mental health conditions managed outside hospital and specialist care.

### Exposure variable

The primary variable of interest was country of birth of the individuals in the study cohort. Individuals were classified according to whether they were born inside or outside the Nordic countries (Sweden, Denmark, Norway, Finland and Iceland). In robustness analyses, we use an alternative measure, capturing whether individuals were born outside a high-income region (Europe, North America, Australia or New Zealand), as well as to capture how long they have been residing in Sweden (more or less than 5 years).

### Outcome variables

We focus on diagnoses and treatments for common perinatal mental health conditions (12 months after childbirth). Relevant diagnoses were identified using ICD-10 codes from primary, specialist and inpatient care. The focus is on capturing common perinatal mental health conditions requiring clinical management: depressive disorders (F32–F33), anxiety disorders (F40–F41), obsessive–compulsive disorder (F42) and puerperal mental disorders (F53). Treatment for common perinatal mental health conditions was defined as receipt of antidepressant medication (Anatomical Therapeutic Chemical Classification System code N06A) and/or psychological therapy identified using KVÅ clinical procedure codes (including cognitive behavioural therapy, interpersonal therapy and other structured psychotherapies).

We define three primary outcomes: case detection, treatment uptake and treatment timeliness. To capture case detection, we focus on new diagnoses for one of the specified conditions within 12 months after childbirth, with no corresponding diagnosis or treatment in the 12 months preceding childbirth. To capture treatment uptake, we measure receipt of treatment (collection of an antidepressant prescription or attending a psychotherapy session) among individuals with a newly recorded mental health diagnosis for one of the specified conditions. To capture timeliness of care, we measure weeks from the first recorded mental health diagnosis to the initiation of treatment (antidepressants or psychotherapy), among individuals who initiated treatment and received a first diagnosis before initiating treatment.

Covariates measured in the year of childbirth included educational attainment (highest level attained), household disposable income, sex of the child and multiple birth status (singleton vs multiple birth).

### Confounders

Mental health outcomes were identified using administrative data and therefore reflect recorded diagnoses and treatments rather than underlying symptom burden. Undiagnosed or untreated conditions are not captured, and observed differences may partly reflect variation in healthcare-seeking or diagnostic practices. Although inclusion of primary care data improves coverage, misclassification remains possible. ‘Newly recorded’ diagnoses were defined using a 12-month look-back window and may include recurrent cases without recent records. In addition, variation in screening practices may influence the likelihood and timing of recorded diagnoses.

### Statistical methods

Analyses were conducted separately for mothers and fathers. We estimated linear regression models throughout. Linear models are used to allow inclusion of municipality fixed effects while retaining the full sample, as non-linear fixed effects models would drop groups with no within-group variation in the outcome. For binary outcomes (case detection and treatment uptake), coefficients can be interpreted as absolute differences in probability (percentage points). For timeliness of treatment, coefficients represent differences in mean weeks from diagnosis to treatment initiation. We do not use Cox regression for this outcome, as time to treatment is fully observed among individuals who initiate treatment; we therefore estimate linear models to obtain mean differences in weeks rather than HRs.

We examine three outcomes: new case detection, treatment uptake (among individuals with a new diagnosis) and timeliness of treatment (among individuals who initiated treatment and were diagnosed before receiving treatment). We present unadjusted models for each outcome, followed by models adjusted for educational attainment, household disposable income, child sex and multiple births. Educational attainment is included as a categorical variable (less than 9 years of schooling; primary; secondary; tertiary of less than 2 years; tertiary of 2 years or more; PhD), with missing values retained as a separate category. Household disposable income in Swedish Krona is also modelled categorically (≤200 000; 200 001–400 000; 400 001–600 000; 600 001–1000 000; >1 000 000), with missing values included as a separate category. No imputation was performed.

All models include calendar year fixed effects. Adjusted models further include municipality fixed effects to account for time-invariant differences across municipalities, with standard errors clustered at the municipality level. An alternative approach would be a multilevel specification with municipality as a higher-level unit. However, as municipality-level factors are likely correlated with individual characteristics, we adopt a fixed effects approach to control for unobserved heterogeneity.

### Patient and public involvement

Patients and members of the public were not involved in the design, conduct, reporting or dissemination plans of this research. The study was based on pseudonymised administrative register data and did not involve direct participant recruitment or contact

## Results

### Descriptive data

Table 1 describes the study population. The analysis includes all parents of first live births in the study regions between 2008 and 2018, comprising 404 043 mothers and 391 068 fathers—reflecting that not all children have a registered father.

**Table 1:** Sample description

	Mothers (n=404 045)	Fathers (n=391 075)
Birth characteristics:		
Female	195 848 (48.5%)	189 679 (48.5%)
Multiple birth	8249 (2.0%)	7961 (2.0%)
Parental characteristics:		
Age at birth	30.9 (5.3)	33.8 (6.2)
Born outside Nordic countries	131 551 (32.6%)	125 432 (32.3%)
Annual household income (Kr)		
Low (≤200k)	70 804 (17.5%)	57 150 (14.6%)
Lower middle (200k–400k)	116 606 (28.9%)	113 232 (28.9%)
Middle (400k–600k)	128 707 (31.8%)	131 210 (33.5%)
Upper middle (600k–1m)	63 244 (15.6%)	65 094 (16.6%)
High (>1m)	12 565 (3.1%)	12 968 (3.3%)
Missing	12 119 (3.0%)	11 421 (2.9%)
Highest achieved education		
<9 years of schooling	12 657 (3.1%)	11 876 (3.0%)
Primary schooling	27 203 (6.7%)	33 974 (8.7%)
Secondary, ≤2 years	29 499 (7.3%)	44 487 (11.4%)
Secondary, >2 years	94 695 (23.4%)	104 061 (26.6%)
Tertiary, ≤2 years	56 858 (14.1%)	57 606 (14.7%)
Tertiary, >2 years	156 373 (38.7%)	110 587 (28.3%)
PhD	4594 (1.1%)	6293 (1.6%)
Missing	22 166 (5.5%)	22 191 (5.7%)

The population covers all individuals with a first live birth in the study regions between 2008 and 2018. Values are n (%) unless otherwise stated. Continuous variables are presented as mean (SD). For mothers, age and immigration background are available for 403 587 individuals. For fathers, age and immigration background are available for 387 690 individuals. Education is only missing for individuals not born in Sweden, for whom educational attainment has not been verified. Income is reported in categories as defined in the analysis (including a missing category for individuals with no taxable income).

### Case detection, treatment and timeliness of care

Key outcomes covering case detection, treatment and timeliness of care are shown in table 2 and reflect recorded diagnoses and treatments rather than underlying prevalence. Among first-time mothers (n=404 045), 7.2% had a newly recorded mental health diagnosis or treatment within 12 months postpartum, compared with 8.0% when including all recorded diagnoses or treatments. Among those with a newly recorded diagnosis, 68.2% received treatment (n=21 984), with 19.3% receiving psychotherapy, 58.4% receiving antidepressants and 9.4% receiving both. Among treated mothers, the mean time from diagnosis to treatment was 5.99 weeks (SD 10.18; n=12 448).

**Table 2:** Summary of key outcomes

	Mothers	Fathers
New mental health diagnosis or treatment	28 895 (7.2%)	17 720 (4.5%)
Conditional on diagnosis		
Received any treatment	15 002 (68.2%)	7490 (69.5%)
Conditional on treatment		
Weeks from diagnosis to treatment	5.99 (10.18)	3.69 (7.85)

The table reports summary statistics for key outcomes across the perinatal mental healthcare pathway. The population includes all first-time parents with a live birth between January 2008 and December 2018 in the study regions. Values are n (%) unless otherwise stated. A new mental health issue is defined as a diagnosis or treatment for a specified mental disorder recorded within 12 months after childbirth, with no corresponding diagnosis or treatment in the 12 months preceding childbirth. Treatment uptake is defined as receipt of antidepressants and/or psychotherapy within 12 months after childbirth among individuals with a diagnosis for one of the relevant mental health issues. Timeliness of care is measured as weeks from first recorded diagnosis within 12 months after childbirth to initiation of treatment (antidepressants or psychotherapy), among individuals who receive treatment and have a diagnosis recorded prior to treatment. Weeks are reported as mean (SD).

Among first-time fathers (n=391 075), 4.5% had a newly recorded mental health diagnosis or treatment, compared with 4.9% when including all recorded diagnoses or treatments. Among those with a diagnosis, 69.5% received treatment (n=10 774), including 19.5% receiving psychotherapy, 60.2% receiving antidepressants and 9.8% receiving both. Among treated fathers, the mean time from diagnosis to treatment was 3.69 weeks (SD 7.85; n=5750).

### Case detection

Column (1) of table 3 presents immigration-related inequalities in case detection. Unadjusted estimates indicate lower probabilities of having a newly recorded mental health issue among individuals born outside the Nordic countries. In adjusted models, controlling for education, income, child sex and multiple births, the probability of case detection was 5.3 percentage points lower for mothers (95% CI −5.8 to −4.9), corresponding to a 59% lower probability relative to Nordic-born mothers. For fathers born outside the Nordic countries, probability of detection was 2.5 percentage points lower (95% CI −2.8 to −2.3), corresponding to a 48% lower probability. Overall, disparities in case detection were large and more pronounced among mothers than fathers.

**Table 3:** Socioeconomic inequities in the perinatal mental health (MH) pathway

	(1)	(2)	(3)
	Detection	Treatment uptake	Timelines
	New MH issue diagnosed or treated	Conditional on new MH diagnosis	Weeks from diagnosis to treatment
Panel A: mothers			
Nordic-born mean	9.0	70.4	6.06
Unadjusted estimates	−5.6 (−5.8 to −5.5)	−12.9 (−14.6 to −11.3)	−0.49 (−1.00 to 0.02)
Adjusted estimates (education, income, sex, multiple births, municipality fixed effects)	−5.3 (−5.8 to −4.9)	−12.1 (−14.2 to −9.9)	−0.51 (−0.96 to −0.06)
Observations	403 587	21 984	12 448
Panel B: fathers			
Nordic-born mean	5.2	71.8	3.52
Unadjusted estimates	−2.2 (−2.4 to −2.1)	−10.2 (−12.2 to −8.1)	0.82 (0.32 to 1.33)
Adjusted estimates (education, income, sex, multiple births, municipality fixed effects)	−2.5 (−2.8 to −2.3)	−7.6 (−10.5 to −4.6)	0.50 (−0.09 to 1.09)
Observations	387 690	10 774	5750

The table reports results for parents of first live-born children between January 2008 and December 2018. All estimates capture inequalities by immigration background (born outside vs inside the Nordic countries). Column (1) shows inequalities in new MH diagnoses, defined as a relevant diagnosis recorded within 12 months after childbirth with no corresponding diagnosis or treatment in the 12 months preceding childbirth. Column (2) shows inequalities in treatment uptake (receipt of antidepressants and/or psychotherapy) conditional on having a diagnosis within 12 months after childbirth. Column (3) shows inequalities in timeliness of care, measured as weeks from first diagnosis within 12 months after childbirth to first treatment (antidepressants or psychotherapy), conditional on receiving treatment and receiving a relevant diagnosis before receiving first treatment. Panel A reports results for mothers; Panel B reports results for fathers. Estimates are from linear models. Coefficients in columns (1) and (2) are risk differences in percentage points; column (3) reports differences in weeks. All models include year fixed effects. Adjusted specifications control for parental education, income, sex of the child, multiple births as well as municipality (kommun) fixed effects, with standard errors clustered at the municipality (kommun) level. Nordic-born means are reported for the relevant estimation sample in each column.

### Treatment uptake: immigration-related inequalities

Column (2) of table 3 presents immigration-related inequalities in treatment uptake. Unadjusted estimates indicate lower probabilities of receiving treatment among immigrant parents. In adjusted models, conditional on a newly recorded mental health diagnosis, treatment uptake was 12.1 percentage points lower among mothers born outside the Nordic countries (95% CI −14.2 to −9.9), corresponding to a 17% lower probability relative to Nordic-born mothers. For fathers born outside the Nordic countries, treatment uptake was 7.6 percentage points lower (95% CI −10.5 to −4.6), corresponding to an 11% lower probability relative to Nordic-born fathers. These results indicate that immigration-related inequalities persist at the treatment stage, including among parents with a formally recorded diagnosis.

### Timeliness of treatment: immigration-related inequalities

Column (3) of table 3 presents immigration-related differences in timeliness of care, measured as weeks from diagnosis to treatment. Unadjusted estimates suggest small differences across groups. In adjusted models, immigrant mothers experienced slightly shorter times from diagnosis to treatment (−0.51 weeks; 95% CI −0.96 to −0.06), while estimates for fathers were small and not statistically significant (0.50 weeks; 95% CI −0.09 to 1.09). Although mothers born outside the Nordic countries were diagnosed later relative to birth (online supplemental table S1), overall differences in timeliness of care were modest.

### Supplementary analyses

Sensitivity analyses using an alternative definition of immigration background (born outside high-income world regions) yielded similar results (online supplemental table S2), with comparable inequalities in case detection and treatment uptake and few differences in timeliness. These findings suggest that inequalities are not confined to or concentrated in the most disadvantaged migrant groups.

In analyses restricted to foreign-born parents, recent arrivals (≤5 years in Sweden) had lower detection and treatment uptake than longer-term migrants (online supplemental table S3), which may reflect factors such as limited familiarity with the healthcare system, language barriers or reduced social support. Among those who initiated treatment, however, recent arrivals experienced shorter times from diagnosis to treatment, particularly among mothers.

Models including facility fixed effects (among those diagnosed) showed similar results, although inequalities in treatment uptake were slightly attenuated (online supplemental table S4).

In a robustness analysis, we additionally included N05B medications (anxiolytics, hypnotics and sedatives) in the treatment definition, rather than restricting the definition to antidepressants (N06A). These medications were not included in the primary specification because they are less specific to common perinatal mental disorders and may also reflect treatment for sleep problems or transient distress. Inclusion of N05B medications yielded substantively similar results (online supplemental table S5).

## Discussion

We document immigration-related inequalities across the perinatal mental healthcare pathway, particularly in case detection and treatment uptake. Parents born outside the Nordic countries were less likely to have a mental health issue recorded in the 12 months following childbirth and, conditional on recorded need, were less likely to receive treatment. Differences in timeliness of care were small and less consistent. Subgroup analyses showed similar patterns under alternative definitions of immigration background. Among foreign-born parents, recent arrivals had lower detection and treatment uptake.

The largest immigration-related inequalities were observed at the stage of case detection (59% lower among mothers and 48% lower among fathers relative to Nordic-born parents). As detection reflects recorded diagnoses rather than underlying prevalence, and survey evidence indicates equal or higher levels of symptoms among immigrant women,^5^ the observed differences among mothers are unlikely to reflect lower need. Comparable evidence is not available for immigrant fathers. Overall, the findings are consistent with inequalities arising through multiple mechanisms. These may include healthcare system factors, such as differences in clinical recognition, communication and screening practices,^7 16^ as well as patient-related factors, including differences in mental health literacy, healthcare-seeking behaviour, treatment preferences and language barriers.^17 18^ Our data do not allow us to distinguish between these potential mechanisms.

We also observe immigration-related inequalities once perinatal mental health issues have been detected. These patterns can be understood in relation to Levesque’s conceptual framework of access to healthcare, which treats access as a process spanning the identification of need, care seeking, reaching services, obtaining care and having needs met.^19^ Conditional on having a newly recorded mental health issue, treatment uptake was 17% lower among immigrant mothers and 11% lower among immigrant fathers. Models including facility fixed effects yielded similar results, although the differences were somewhat attenuated, suggesting that the observed inequalities are not explained solely by differences in the healthcare facilities attended. Differences in timeliness of care were comparatively modest and not consistently observed across groups. This may reflect that there is less scope for variation in the timing of treatment once individuals have entered the care pathway than in earlier stages, such as case detection and treatment initiation. For example, providers may differ more in decisions about whether to diagnose or initiate treatment than in the timing of treatment following diagnosis, while patients may face greater barriers to seeking care or initiating treatment than to the timing of treatment once a diagnosis has been made. Alternatively, the comparatively small differences in timeliness may reflect selection into the treated population, as this analysis is restricted to individuals who received treatment. Although the data do not permit identification of specific mechanisms, gaps may reflect barriers at different stages of access, including treatment acceptance, access to linguistically or culturally appropriate services, appointment attendance, prescribing behaviour or collection of prescriptions.

This study has several strengths. We used linked national and regional registers with near-complete coverage of the study population and included detailed individual characteristics and data from all levels of care. Previous work has primarily used data from tertiary and specialist consultations, and area-based measures of socioeconomic status. Furthermore, by examining sequential stages of the care pathway, we move beyond single-endpoint analyses and provide a more granular assessment of where inequalities arise.

This study has several limitations. Mental health outcomes were identified from administrative data and therefore reflect recorded diagnoses and dispensed drugs rather than underlying disease prevalence. In addition, ‘new’ mental health issues were defined based on the absence of recorded diagnosis or treatment in the 12 months prior to childbirth; this may misclassify recurrent or chronic conditions with earlier episodes outside this window. We capture treatment uptake based on dispensed prescriptions and recorded psychotherapy contacts and therefore cannot assess actual medication use or engagement with therapy. In addition, we cannot distinguish between provider-related and patient-related factors underlying lower treatment uptake, as we do not observe whether treatment was offered but declined or medication was prescribed but not dispensed. A further limitation is that parents born outside the Nordic countries comprise a heterogeneous population. Although we explored heterogeneity by broad world region and timing of arrival, future studies should investigate differences by region of origin, refugee status and language background. Finally, we lacked information on symptom severity, language proficiency and treatment preferences, limiting our ability to identify the mechanisms underlying observed disparities.

The central contribution of this study is to show that immigration-related inequalities persist across successive stages of the perinatal mental healthcare pathway. Immigrant parents are less likely to have mental health problems recorded and, conditional on need, less likely to receive treatment. These findings suggest that reducing inequalities requires coordinated interventions across the care pathway, including equitable implementation of postnatal screening and improved follow-up after identification. Further research is needed to identify effective strategies to improve access to and delivery of perinatal mental healthcare among immigrant populations.

## Supplementary material

10.1136/bmjopen-2026-122722 online supplemental file 1

## Data Availability

Data may be obtained from a third party and are not publicly available.
