# Supplementary material for: Immigration-related inequalities across the perinatal mental health care pathway among new parents in Sweden: a population-based cohort study
Source: BMJ Open. 2026 Aug 17;16(8):e122722. doi: 10.1136/bmjopen-2026-122722 (PMC13504490; doi:10.1136/bmjopen-2026-122722)
Supplement: Supplementary file 1 [file bmjopen-2026-122722.reviewer_comments.pdf]

PEER REVIEW HISTORY

BMJ Open publishes all reviews undertaken for accepted manuscripts. Reviewers are asked to complete a checklist review form and are provided with free text boxes to elaborate on their assessment. These free text comments are reproduced below.

ARTICLE DETAILS

Title (Provisional)

Immigration-related inequalities across the perinatal mental health care pathway among new parents in Sweden: a population-based cohort study

Authors

Kovacs, Roxanne; Ng, Nawi; Kjellsson, Gustav

VERSION 1 - REVIEW

**Reviewer**	**1**
**Name**	**Nigatu, Desalegn**
**Affiliation**	**Mettu University, Nursing**
**Date**	**07-Jun-2026**
**COI**	**None**

Topic: Immigration-related inequalities across the perinatal mental health care pathway among new parents in Sweden: a population-based cohort study.

- The authors attribute the lower rates of case detection and treatment uptake among immigrant parents primarily to barriers within the healthcare system. While this interpretation is plausible, the discussion would be strengthened by a more balanced consideration of alternative explanations, such as differences in mental health literacy, cultural perceptions of mental illness, stigma, treatment preferences, and healthcare-seeking behaviors. To what extent might these factors contribute to the observed disparities?

Specific Questions

1. **Case Detection**

   The manuscript states that lower detection rates are unlikely to reflect lower need because survey evidence indicates equal or higher symptom levels among immigrant women. Were similar data available for fathers? If not, the interpretation for fathers should be made more cautiously.

2. **Heterogeneity Among Immigrant Groups**

The category "parents born outside the Nordic countries" is highly heterogeneous. Did the authors explore variations by region of origin, refugee status, language background, or socioeconomic characteristics? Such analyses could provide important insights into the mechanisms underlying the observed inequalities.

3. **Recent Arrivals**

   The finding that recent arrivals had lower detection and treatment uptake is noteworthy. Can the authors elaborate on whether this may reflect limited familiarity with the healthcare system, language barriers, lack of social support, or differences in eligibility for healthcare services?

4. **Treatment Uptake**

   Treatment uptake was lower among immigrant parents after detection. Is it possible to distinguish whether this reflects provider-related barriers (e.g., referral practices, availability of culturally adapted services) versus patient-related factors (e.g., treatment acceptance, perceived need, or stigma)?

5. **Timeliness of Care**

   The discussion notes that differences in timeliness were small and inconsistent. Could the authors provide potential explanations for why inequalities were pronounced in detection and treatment uptake but not in timeliness once individuals entered the care pathway?

**Reviewer**	**2**
**Name**	**Beckett, Cynthia**
**Affiliation**	**The Ohio State University College of Nursing, Nursing**
**Date**	**16-Jul-2026**
**COI**	**None**

Thank you for your submission. Very interesting cohort study. This is an issue that is globally important.

VERSION 1 - AUTHOR RESPONSE

Reviewer 1

General comment:

The authors attribute the lower rates of case detection and treatment uptake among immigrant parents primarily to barriers within the healthcare system. While this interpretation is plausible, the discussion would be strengthened by a more balanced

consideration of alternative explanations, such as differences in mental health literacy, cultural perceptions of mental illness, stigma, treatment preferences, and healthcare-seeking behaviors. To what extent might these factors contribute to the observed disparities?

Response: We thank the reviewer for this thoughtful comment. We agree that the observed inequalities are likely to reflect a range of interacting mechanisms and that barriers within the healthcare system represent only one possible explanation. We have revised the Discussion to provide a more balanced interpretation by acknowledging that differences in mental health literacy, healthcare-seeking behaviours, language barriers, treatment preferences and other patient-related factors may also contribute to the observed disparities. We also clarify that our data do not allow us to distinguish between these potential mechanisms.

Changes made in manuscript: We revised the Discussion to provide a more balanced interpretation of the observed inequalities. Specifically, we now acknowledge that the findings may reflect a combination of healthcare system factors (e.g. clinical recognition, communication and screening practices) and patient-related factors (e.g. mental health literacy, healthcare-seeking behaviour, treatment preferences and language barriers), and we clarify that our data do not allow us to distinguish between these potential mechanisms.

1. Case Detection

Reviewer comment:

The manuscript states that lower detection rates are unlikely to reflect lower need because survey evidence indicates equal or higher symptom levels among immigrant women. Were similar data available for fathers? If not, the interpretation for fathers should be made more cautiously.

Response: We thank the reviewer for this helpful comment. We agree that the evidence regarding underlying need is stronger for mothers than for fathers. While there is substantial evidence that immigrant women experience similar or higher levels of perinatal mental health symptoms than native-born women, comparable evidence for fathers is limited. We have therefore revised the manuscript to interpret the findings for fathers more cautiously.

Changes made in manuscript: In the discussion (page 10/11) we now state: The largest immigration-related inequalities were observed at the stage of case detection (59% lower among mothers and 48% lower among fathers relative to Nordic-born parents). As detection reflects recorded diagnoses rather than underlying prevalence, and survey evidence indicates equal or higher levels of symptoms among immigrant

women [5], the observed differences among mothers are unlikely to reflect lower need. Comparable evidence is not available for immigrant fathers.

2. Heterogeneity Among Immigrant Groups

Reviewer comment:

The category 'parents born outside the Nordic countries' is highly heterogeneous. Did the authors explore variations by region of origin, refugee status, language background, or socioeconomic characteristics?

Response: We thank the reviewer for this important comment and fully agree that the population of parents born outside the Nordic countries is highly heterogeneous. We considered more detailed analyses by region of origin; however, many origin groups were relatively small, resulting in limited statistical power and imprecise estimates. Information on refugee status and language background was not available in our data. Individual socioeconomic characteristics, including educational attainment and household income, were included as covariates in all adjusted analyses. To explore heterogeneity where feasible, we conducted two additional analyses: one using an alternative classification distinguishing individuals born outside high-income world regions (Appendix Table A2), which yielded similar patterns to the main analysis, and another examining duration of residence in Sweden among foreign-born parents (Appendix Table A3), which showed lower detection and treatment uptake among recent arrivals than longer-term migrants. In response to the reviewer's suggestion, we have also expanded the Discussion to explicitly acknowledge the heterogeneity of the immigrant population as a limitation of our exposure definition and note that future studies should investigate differences by region of origin, refugee status and other migration-related characteristics.

Changes made in manuscript:

We revised the Discussion (page 11) to clarify that, although analyses by region of origin were not feasible because of limited sample sizes for many groups, we explored heterogeneity using two alternative approaches. Analyses using a broader classification based on high-income world regions yielded similar results to the main analysis, while analyses by duration of residence showed lower detection and treatment uptake among recent arrivals than longer-term migrants. Future studies should investigate differences by region of origin, refugee status and language background.

3. Recent Arrivals

Reviewer comment:

Can the authors elaborate on whether this may reflect limited familiarity with the healthcare system, language barriers, lack of social support, or differences in eligibility for healthcare services

Response: We thank the reviewer for this helpful suggestion. We agree that the lower detection and treatment uptake observed among recent arrivals may reflect factors such as limited familiarity with the healthcare system, language barriers or reduced social support, although our data do not allow us to distinguish between these potential mechanisms. As shown in the supplementary analysis (Appendix Table A3), recent arrivals who initiated treatment experienced similar or shorter times from diagnosis to treatment, particularly among mothers. This finding may indicate that barriers arise earlier in the care pathway, but it may also reflect selection into the treated population, for example if those receiving treatment had greater clinical need. Our data do not allow us to distinguish between these explanations. We have added this discussion to the description of the supplementary analysis (Appendix Table A3).

Changes made in manuscript: We expanded the description of the supplementary analysis (Appendix Table A3) to note that the lower detection and treatment uptake observed among recent arrivals may reflect factors such as limited familiarity with the healthcare system, language barriers or reduced social support. We also clarified that, among those who initiated treatment, recent arrivals experienced similar or shorter times from diagnosis to treatment, and that the available data do not allow us to distinguish between the potential explanations for this finding.

4. Treatment Uptake

Reviewer comment:

Is it possible to distinguish whether lower treatment uptake reflects provider-related barriers versus patient-related factors?

Response: We thank the reviewer for this important comment. Unfortunately, our data do not allow us to distinguish between provider-related and patient-related mechanisms underlying the lower treatment uptake observed among immigrant parents. We estimated additional models including facility fixed effects (Appendix Table A4), which yielded similar results, although inequalities in treatment uptake were somewhat attenuated. This suggests that the observed disparities are not explained solely by differences in the healthcare facilities attended. However, even within facilities, our data do not allow us to distinguish between provider-related and patient-related mechanisms. While we observe recorded psychotherapy contacts and dispensed prescriptions, we do not observe treatment offers, treatment refusal, or

prescriptions that were never dispensed. We have clarified these limitations in the Discussion.

Changes made in manuscript: The discussion (page 11) now notes: In addition, we cannot distinguish between provider-related and patient-related factors underlying lower treatment uptake, as we do not observe whether treatment was offered but declined or medication was prescribed but not dispensed.

It also notes: Conditional on having a newly recorded mental health issue, treatment uptake was 17% lower among immigrant mothers and 11% lower among immigrant fathers. Models including facility fixed effects yielded similar results, although the differences were somewhat attenuated, suggesting that the observed inequalities are not explained solely by differences in the healthcare facilities attended.

5. Timeliness of Care

Reviewer comment:

Could the authors explain why inequalities were pronounced in detection and treatment uptake but not in timeliness once individuals entered the care pathway?

Response: We thank the reviewer for this helpful comment. Our data do not allow us to distinguish between the underlying mechanisms. One possible explanation is that there is less scope for variation in the timing of treatment once individuals have entered the care pathway than in earlier stages, such as case detection or treatment initiation. For example, providers may differ more in decisions about whether to diagnose or initiate treatment than in the timing of treatment once a diagnosis has been made. Similarly, patients may face greater barriers to seeking care or initiating treatment than to the timing of treatment following diagnosis. An alternative explanation is selection into the treated population, as this analysis is restricted to individuals who received treatment. We have added a brief discussion of these possible explanations to the manuscript.

Changes made in manuscript: The discussion (page 11) now reads: This may reflect that there is less scope for variation in the timing of treatment once individuals have entered the care pathway than in earlier stages, such as case detection and treatment initiation. For example, providers may differ more in decisions about whether to diagnose or initiate treatment than in the timing of treatment following diagnosis, while patients may face greater barriers to seeking care or initiating treatment than to the timing of treatment once a diagnosis has been made. Alternatively, the comparatively small differences in timeliness may reflect selection into the treated population, as this analysis is restricted to individuals who received treatment.

Reviewer 2

Comment:

Thank you for your submission. Very interesting cohort study. This is an issue that is globally important. Clearly presented. Nicely done.

Response:

Thank you.
