# Supplementary material for: Immigration-related inequalities across the perinatal mental health care pathway among new parents in Sweden: a population-based cohort study
Source: BMJ Open. 2026 Aug 17;16(8):e122722. doi: 10.1136/bmjopen-2026-122722 (PMC13504490; doi:10.1136/bmjopen-2026-122722)
Supplement: Supplementary file 2 [file bmjopen-16-8-s001.docx]

**Supplemental Material – Immigration-related inequalities across the perinatal mental health care pathway among new parents in Sweden: a population-based cohort study**

### A1 Robustness analysis on timeliness of care

Table S1: Socio-economic inequities in timeliness of care

|  | (1) |
| --- | --- |
|  | Weeks from birth to diagnosis |
| **Panel A: Mothers** |  |
| Nordic-born mean | 19.56 |
| Unadjusted estimates | 1.02 (0.41 to 1.64) |
| Adjusted estimates (education, income, sex, multiple births, municipality fixed effects) | 0.91 (0.29 to 1.53) |
| Observations | 18,649 |
| **Panel B: Fathers** |  |
| Nordic-born mean | 22.14 |
| Unadjusted estimates | 0.19 (−0.56 to 0.95) |
| Adjusted estimates (education, income, sex, multiple births, municipality fixed effects) | 0.52 (−0.19 to 1.22) |
| Observations | 9,210 |

Notes: The table reports results for parents of first live-born children between January 2008 and December 2018. All estimates capture inequalities by immigration background (born outside vs inside the Nordic countries). Column (1) shows inequalities in timeliness of care, measured as weeks from childbirth to a first relevant diagnosis. Panel A reports results for mothers; Panel B reports results for fathers. Estimates are from linear models. Coefficients show differences in weeks. All models include year fixed effects. Adjusted specifications control for parental education, income, sex of the child, multiple births as well as municipality (kommun) fixed effects, with standard errors clustered at the municipality (kommun) level. Nordic-born means are reported for the relevant estimation sample in each column.

### A2 Robustness analysis capturing individuals born outside high-income world regions (Europe, North America, Australia and New Zealand)

Table S2: Socio-economic inequities in the perinatal mental health pathway in high-income world regions

|  | (1) | (2) | (3) |
| --- | --- | --- | --- |
|  | Detection | Treatment uptake | Timelines |
|  | New MH issue diagnosed or treated | Conditional on new MH diagnosis | Weeks from diagnosis to treatment |
| **Panel A: Mothers** |  |  |  |
| High-income region mean | 8.2 | 69.7 | 6.03 |
| Unadjusted estimates | -5.2 (-5.4 to -5.0) | -14.2 (-16.2 to -12.2) | -0.46 (-1.11 to 0.18) |
| Adjusted estimates (education, income, sex, multiple births, municipality fixed effects) | -4.4 (-5.5 to -3.3) | -12.5 (-15.2 to -9.9) | -0.60 (-1.27 to 0.08) |
| Observations | 403,587 | 21,984 | 12,448 |
| **Panel B: Fathers** |  |  |  |
| High-income region mean | 4.9 | 70.9 | 3.62 |
| Unadjusted estimates | -2.0 (-2.1 to -1.8) | -10.3 (-12.9 to -7.8) | 0.55 (-0.07 to 1.18) |
| Adjusted estimates (education, income, sex, multiple births, municipality fixed effects) | -2.1 (-2.6 to -1.6) | -6.7 (-9.6 to -3.9) | 0.06 (-0.50 to 0.62) |
| Observations | 387,690 | 10,774 | 5,750 |

Notes: The table reports results for parents of first live-born children between January 2008 and December 2018. All estimates capture inequalities by immigration background (born outside vs inside a high-income region: Europe, North America, Australia and New Zealand). Column (1) shows inequalities in new mental health (MH) diagnoses, defined as a relevant diagnosis recorded within 12 months after childbirth with no corresponding diagnosis or treatment in the 12 months preceding childbirth. Column (2) shows inequalities in treatment uptake (receipt of antidepressants and/or psychotherapy) conditional on having a diagnosis within 12 months after childbirth. Column (3) shows inequalities in timeliness of care, measured as weeks from first diagnosis within 12 months after childbirth to first treatment (antidepressants or psychotherapy), conditional on receiving treatment and receiving a relevant diagnosis before receiving first treatment. Panel A reports results for mothers; Panel B reports results for fathers. Estimates are from linear models. Coefficients in columns (1)–(2) are risk differences in percentage points; column (3) reports differences in weeks. All models include year fixed effects. Adjusted specifications control for parental education, income, sex of the child, multiple births as well as municipality (kommun) fixed effects, with standard errors clustered at the municipality (kommun) level. Nordic-born means are reported for the relevant estimation sample in each column.

### A3 Robustness analysis focusing on recent arrivals

Table S3: Inequities in the perinatal mental health pathway among foreign-born individuals (based on arrival date)

|  | (1) | (2) | (3) |
| --- | --- | --- | --- |
|  | Detection | Treatment uptake | Timelines |
|  | New MH issue diagnosed or treated | Conditional on new MH diagnosis | Weeks from diagnosis to treatment |
| **Panel A: Mothers** |  |  |  |
| Non-recent arrival mean | 5.8 | 60.0 | 5.93 |
| Unadjusted estimates | -4.0 (-4.2 to -3.8) | -9.6 (-13.3 to -6.0) | -1.54 (-2.62 to -0.45) |
| Adjusted estimates (education, income, sex, multiple births, municipality fixed effects) | -2.9 (-3.2 to -2.7) | -7.2 (-13.2 to -1.2) | -1.79 (-3.22 to -0.35) |
| Observations | 126,135 | 3,669 | 1,795 |
| **Panel B: Fathers** |  |  |  |
| Non-recent arrival mean | 4.5 | 63.1 | 4.51 |
| Unadjusted estimates | -2.9 (-3.1 to -2.7) | -7.0 (-11.6 to -2.4) | -0.97 (-2.15 to 0.21) |
| Adjusted estimates (education, income, sex, multiple births, municipality fixed effects) | -2.1 (-2.5 to -1.8) | -6.6 (-10.8 to -2.4) | -0.91 (-2.21 to 0.38) |
| Observations | 121,670 | 2,435 | 1,176 |

Notes: The table reports results for parents of first live-born children between January 2008 and December 2018. The analysis is restricted to foreign-born individuals (born outside the Nordic countries). Estimates compare recent arrivals (defined as residing in Sweden for less than five years prior to childbirth) with longer-term migrants. Column (1) shows inequalities in new mental health (MH) diagnoses, defined as a relevant diagnosis recorded within 12 months after childbirth with no corresponding diagnosis or treatment in the 12 months preceding childbirth. Column (2) shows inequalities in treatment uptake (receipt of antidepressants and/or psychotherapy) conditional on having a diagnosis within 12 months after childbirth. Column (3) shows inequalities in timeliness of care, measured as weeks from first diagnosis within 12 months after childbirth to first treatment (antidepressants or psychotherapy), conditional on receiving treatment and receiving a relevant diagnosis before receiving first treatment. Panel A reports results for mothers; Panel B reports results for fathers. Estimates are from linear models. Coefficients in columns (1)–(2) are risk differences in percentage points; column (3) reports differences in weeks. All models include year fixed effects. Adjusted specifications control for parental education, income, sex of the child, multiple births as well as municipality (kommun) fixed effects, with standard errors clustered at the municipality (kommun) level. Nordic-born means are reported for the relevant estimation sample in each column.

### A4 Robustness analysis including facility fixed effects

Table S4: Inequities in the treatment uptake and timeliness of care (including facility fixed effects)

|  | (1) | (2) |
| --- | --- | --- |
|  | Treatment uptake | Timelines |
|  | Conditional on new MH diagnosis | Weeks from diagnosis to treatment |
| **Panel A: Mothers** |  |  |
| Nordic-born mean | 70.4 | 6.06 |
| Unadjusted estimates | -12.9 (-14.6 to -11.3) | -0.49 (-1.00 to 0.02) |
| Adjusted estimates (education, income, sex, multiple births, facility fixed effects) | -10.6 (-12.5 to -8.6) | -0.38 (-0.91 to 0.16) |
| Observations | 21,984 | 12,448 |
| **Panel B: Fathers** |  |  |
| Nordic-born mean | 71.8 | 3.52 |
| Unadjusted estimates | -10.2 (-12.2 to -8.1) | 0.82 (0.32 to 1.33) |
| Adjusted estimates (education, income, sex, multiple births, facility fixed effects) | -6.2 (-8.9 to -3.5) | 0.80 (-0.02 to 1.63) |
| Observations | 10,774 | 5,750 |
|  | Yes | Yes |

Notes: The table reports results for parents of first live-born children between January 2008 and December 2018. All estimates capture inequalities by immigration background (born outside vs inside the Nordic countries). Column (1) shows inequalities in treatment uptake (receipt of antidepressants and/or psychotherapy) conditional on having a diagnosis within 12 months after childbirth. Column (2) shows inequalities in timeliness of care, measured as weeks from first diagnosis within 12 months after childbirth to first treatment (antidepressants or psychotherapy), conditional on receiving treatment and receiving a relevant diagnosis before receiving first treatment. Panel A reports results for mothers; Panel B reports results for fathers. Estimates are from linear models. Coefficients in columns (1) are risk differences in percentage points; column (2) reports differences in weeks. All models include year fixed effects. Adjusted specifications include facility fixed effects and control for parental education, income, sex of the child, multiple births, with standard errors clustered at the municipality (kommun) level. Nordic-born means are reported for the relevant estimation sample in each column.

### A5 Robustness analysis including N05B medications

Table S5: Robustness analysis including N05B medications in the definition of perinatal mental health treatment

|  | (1) |
| --- | --- |
|  | Detection |
|  | New MH issue diagnosed or treated |
| **Panel A: Mothers** |  |
| Nordic-born mean | 9.8 |
| Unadjusted estimates | -5.9 (-6.1 to -5.7) |
| Adjusted estimates (education, income, sex, multiple births, municipality fixed effects) | -5.5 (-6.0 to -5.1) |
| Observations | 403,587 |
| **Panel B: Fathers** |  |
| Nordic-born mean | 6.1 |
| Unadjusted estimates | -2.3 (-2.4 to -2.1) |
| Adjusted estimates (education, income, sex, multiple births, municipality fixed effects) | -2.6 (-2.9 to -2.4) |
| Observations | 387,690 |

Notes: The table reports results for parents of first live-born children between January 2008 and December 2018. All estimates capture inequalities by immigration background (born outside vs inside the Nordic countries). Column (1) shows inequalities in new mental health (MH) diagnoses, defined as a relevant diagnosis recorded within 12 months after childbirth with no corresponding diagnosis or treatment (N06A or N05B) in the 12 months preceding childbirth. All models include year fixed effects. Adjusted specifications control for parental education, income, sex of the child, multiple births as well as municipality (kommun) fixed effects, with standard errors clustered at the municipality (kommun) level. Nordic-born means are reported for the relevant estimation sample in each column.
